# Serum 25-hydroxy vitamin D levels in age-related macular degeneration

**DOI:** 10.1186/s40942-022-00368-2

**Published:** 2022-03-07

**Authors:** Antonio Pérez Serena, Daisy Paola Martínez Betancourt, Fernando González del Valle, José M. Ruiz-Moreno

**Affiliations:** 1 Department of Ophthalmology, Hospital General La Mancha Centro, Ciudad Real, Alcázar de San Juan, Spain; 2 Department of Ophthalmology, Hospital Universitario Juan Ramón Jiménez, Ronda Exterior Norte s/n, 21005 Huelva, Spain; 3 Department of General Medicine, Centro de Salud Trigueros, Trigueros, Huelva, Spain; 4 Universidad Castilla-La Mancha, Albacete, Spain; 5 Department of Ophthalmology, Hospital Universitario Puerta de Hierro-Majadahonda, Madrid, Spain

**Keywords:** Vitamin D, Age-related macular degeneration, 25-hydroxy vitamin D, Vitamin D deficiency, 25(OH)D

## Abstract

**Background:**

The aim of this study was to determine the 25-hydroxy vitamin D (25(OH)D) levels in age-related macular degeneration (AMD) patients.

**Methods:**

Age-related macular degeneration (AMD) patients were classified into four groups: early AMD (N = 10), intermediate AMD (N = 12), advanced atrophic AMD (N = 19) and advanced neovascular AMD (N = 52) after undergoing fundus photography. Serum 25(OH)D levels of all subjects were evaluated. From a random control group of 326 patients whose 25(OH)D levels had been measured, a group of 93 were selected to match the age range of the AMD group. We measured 25(OH)D levels during the same period to rule out seasonal variation.

**Results:**

A total of 93 AMD patients (36 males and 57 females) and 93 healthy individuals (39 males and 54 females) were enrolled in this study with the mean age of 78.96 ± 8.46 vs. 78.80 ± 8.35, respectively. The patients affected by AMD had statistically significant lower 25(OH)D levels (15 ± 10 ng/mL) than the healthy subjects control group (21 ± 14 ng/mL) (p = 0.004). However, the median 25(OH)D levels in early AMD, intermediate AMD, advanced atrophic AMD and advanced neovascular AMD (12.5 ± 7.3; 15 ± 11; 15 ± 8 and 17 ± 11.5, respectively) were not statistically significant (p = 0.442).

**Conclusion:**

This study shows that patients affected by AMD had lower vitamin D levels compared to healthy subjects. Further research is necessary to investigate the possible association between 25(OH)D levels and AMD.

## Background

Age-related macular degeneration (AMD) is a complex disease associated with a high risk of complications that affect vision. It develops mainly in people over 50 years of age and a key characteristic is the accumulation of a series of extracellular deposits in the macula, mainly drusen [1]. It is the main cause of irreversible blindness in subjects over 55 years of age in developed countries with millions of affected patients and, in addition, with high potential to rise due to the increase in the life expectancy of the population [2, 3].

AMD has historically been classified into 2 types: dry or non-exudative AMD (dAMD) and wet, exudative, or neovascular AMD (nAMD), dAMD forms the majority of diagnosed cases and nAMD is responsible for the majority of severe vision loss. Although AMD is the major cause of severe vision loss, the geographic atrophy produced by dAMD can also cause significant vision loss [4]. More recently, AMD has been classified according to 3 clinical stages: early AMD, intermediate AMD and advanced AMD divided into atrophic or neovascular AMD [5].

Vitamin D could play a role in the pathophysiology of AMD. Vitamin D is known to be implicated as being a protective factor in certain diseases such as cancer, cardiovascular diseases, bone diseases, kidney disease, and other diseases [6]. Most vitamin D is produced by the skin in the form of vitamin D3 due to the ultraviolet rays from sun exposure, and as such, due to confinement, these levels may have been affected [7]. The main function of vitamin D is the absorption of calcium and phosphorus from the diet, thereby contributing to the mineralization of bone. Osteoporosis, a disease with a deficit in bone mass density, would be a clear example of its association with vitamin D deficiency [8].

Oxidative stress and inflammation lead to photoreceptor degeneration and appear to be involved in the pathophysiology of AMD [9]. Vitamin D appears to increase the expression of antioxidant genes and therefore has the ability to reduce oxidative damage that leads to photoreceptors degeneration [10]. In fact, the vitamin D receptor (VDR) is detected in retinal pigment epithelium and retinal photoreceptor cells [11, 12]. Vitamin D could play a role in reducing chronic oxidative stress, inhibiting amyloid protein deposits, inhibiting chronic inflammation and therefore also reducing angiogenesis [13].

The aim of the present study was to determine 25(OH)D levels in AMD patients.

## Methods

This cross-sectional study was approved by the Hospital General la Mancha Centro Ethics Committee (Alcázar de San Juan, Spain) and was carried out in accordance with the Ethical Principles of the Declaration of Helsinki. All candidates received detailed information about the nature of the investigation, and all provided their written informed consent. This study included patients who had visited Hospital General la Mancha Centro, Alcázar de San Juan (Spain) and Hospital General de Tomelloso, Tomelloso (Spain), from February 2021 to April 2021.

We categorized the participants into 4 AMD groups according to Ferris clinical classification [5] after undergoing fundus photography as follows: early AMD (N = 10), intermediate AMD (N = 12), advanced atrophic AMD (N = 19) and advanced neovascular AMD (N = 52), and those without any macular degeneration (control group).

We excluded patients with osteoporosis, vitamin D absorption problems, chronic renal failure, liver disease or parathyroid disease based on the medical records in our hospital. In addition, we excluded patients who were taking vitamin D supplements.

We also excluded patients with amblyopia, retinal dystrophy, pathologic myopia, diabetic retinopathy, retinal vein occlusion and retinal artery occlusion.

From a group of 326 randomly selected patients whose 25 (OH)D level had been measured, we chose 93 to act as a control group to match the AMD group.

We measured 25(OH)D in the serum of all patients during the same period to rule out seasonal variation of 25(OH)D levels. 25(OH)D was determined by electrochemiluminiscence immunoassay (ECLIA) (Roche cobas e 801).

The optimal vitamin D status is defined in different ways [14, 15]. Plasma 25(OH)D concentrations were assessed from blood samples and categorized as severe deficiency (< 10 ng/mL), deficiency (10–19 ng/mL), insufficiency (20–29 ng/mL), or sufficiency (≥ 30 ng/mL).

### Secondary outcome measures

For all AMD patients the best corrected visual acuity (BCVA), intraocular pressure (IOP), refraction, state of the lens and central foveal thickness (CFT) were measured. The use of AREDS2 supplementation and smoking status were also checked. BCVA was measured with Snellen charts (Topcon cc-100 hw3.0) and converted into logMAR for statistical analysis purposes. The IOP in mm Hg was measured by Goldmann applanation tonometer. Refraction was measured by Canon RK-5 and Topcon Auto kerato-refractometers and was calculated as spherical equivalent. Fundus photography was taken to categorize AMD according to Ferris clinical classification and to measure CFT DRI OCT Triton plus (Topcon) and OCT 3D OCT-2000 (Topcon) were used. We classified the state of the lens as phakic or pseudophakic. We excluded all patients taking AREDS2 supplementation containing vitamin D. Smoking status was defined as a current smoker, ex-smoker, and non-smoker.

Strict classification was used to analyse the affected eye. Where the degree of severity did not differ, the eye with worse BCVA was used as the affected eye.

### Sample size

The number of patients needed was estimated based on the 25(OH)D levels provided by Kan E et al. [16]; with a statistical power of 90% and an α error of 0.05, 140 patients (n = 70 per group) will be needed.

### Statistical analysis

Statistical analysis was performed using the Statistical Package for Social Sciences (IBM SPSS Version 24) for Windows. The distribution of numeric data was assessed by the Kolmogorov–Smirnov test. Pearson’s chi-squared test was employed to examine the differences between categorical variables. The Mann–Whitney U test was used for the comparison the differences between two groups. The Kruskal–Wallis H test was used to analyze the differences between three or more groups. Values of p < 0.05 was considered as statistically significant.

## Results

A total of 93 AMD patients (36 males and 57 females) and 93 healthy individuals (39 males and 54 females) were enrolled in this study with the mean age of 78.96 ± 8.5 and 78.8 ± 8.4, respectively. There was no significant difference between AMD group and control group in terms of age (p = 0.970) and gender (F/M: 61.3%/38.7% Vs 58.1%/41.9%;p = 0.654). Table 1 shows the main characteristics of patients included in our study.

**Table 1:** Main characteristics of patients included in our study

	AMD Group (n = 93)	Control Group (n = 93)	p
Age, mean ± SD	78.96 ± 8.5	78.8 ± 8.4	0.970
Gender			
Female	57 (61.3%)	54 (58.1%)	0.654
Male	36 (38.7%)	39 (41.9%)	
Levels Vit D, median ± IQR	15 ± 10	21 ± 14	0.004
Sufficiency	10 (10.8%)	19 (20.4%)	0.013
Insufficiency	21 (22.6%)	34 (36.6%)	
Deficiency	47 (50.5%)	29 (31.2%)	
Severe deficiency	15 (16.1%)	11 (11.8%)	
Normal	10 (10.8%)	19 (20.4%)	0.069
Pathological	83 (89.2%)	74 (79.6%)	
Sufficiency + insufficiency	31 (33.3%)	53 (57%)	0.001
Deficiency + severe deficiency	62 (66.7%)	40 (43%)	

*SD* standard deviation, *IQR* interquartile range

Of 93 patients with AMD, 10 subjects had early AMD (10.8%), 12 had intermediate AMD (12.9%), 19 had advanced dAMD (20.4%) and 52 had advanced nAMD (55.9%). The mean BCVA, mean IOP, mean CFT was + 1.11 ± 0.95 logMAR, 15.20 ± 2.99 mm Hg and 61.95 ± 63,61 µm, respectively. Of 93 patients with AMD, 49 (52.7%) were phakic and 44 pseudophakic (47.3%). 34 (36.6%) patients were identified to be taking AREDS2 supplementation and 59 (63.4%) were not taking any AREDS2 supplementation. 6 (6.5%) patients were current smokers, 25 (26.9%) were ex-smokers and 62 (66.7%) were non-smokers.

**Fig. 1:**
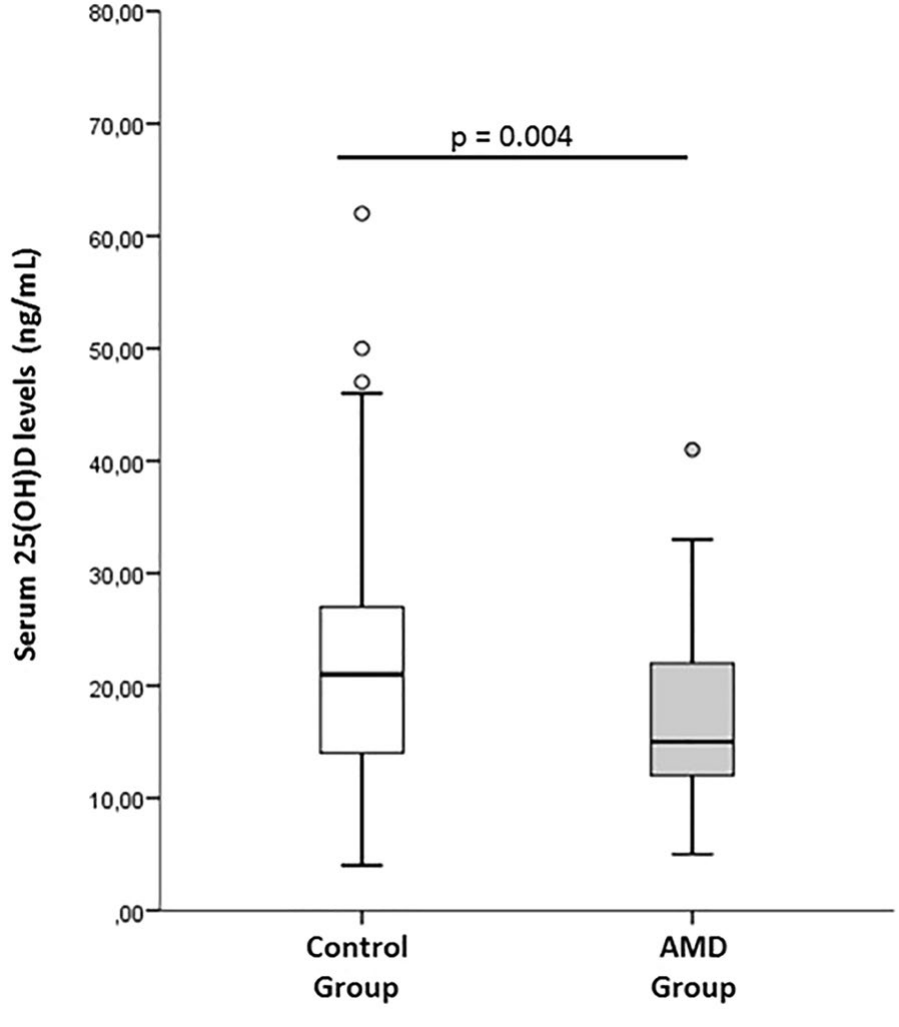
Serum 25(OH)D levels in Control versus AMD patients

The patients affected by AMD had statistically significant lower serum 25(OH)D levels (15 ± 10 ng/mL) than healthy subject group (21 ± 14 ng/mL) (p = 0.004). Fig. 1 and Table 1

**Fig. 2:**
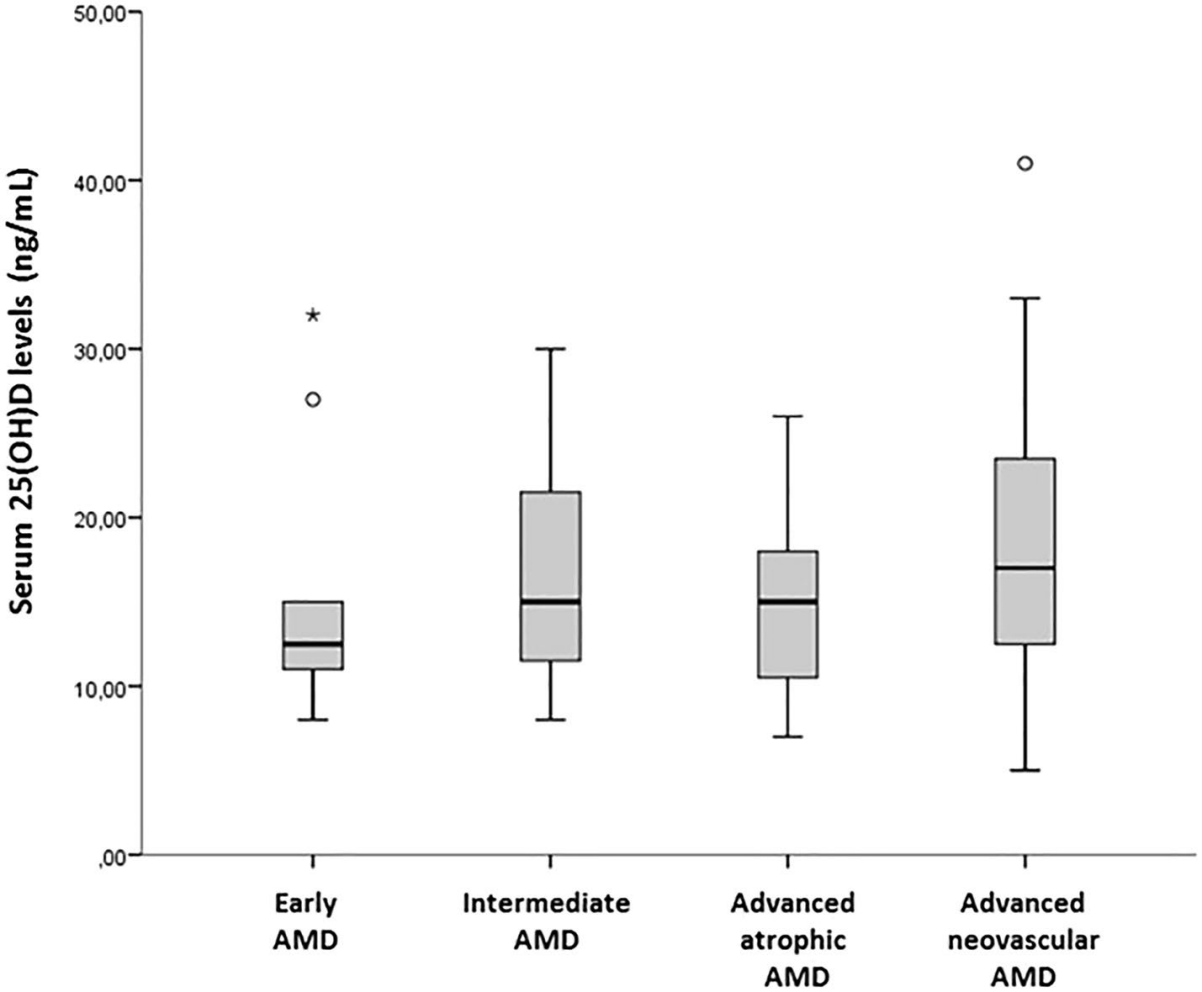
Serum 25(OH)D levels according to AMD Ferris clinical classification

However, the median 25(OH)D levels in early AMD, intermediate AMD, advanced dAMD and advanced nAMD (12.5 ± 7.3, 15 ± 11, 15 ± 8, 17 ± 11.5, respectively) were not statistically significant (p = 0.442). Fig. 2.

**Fig. 3:**
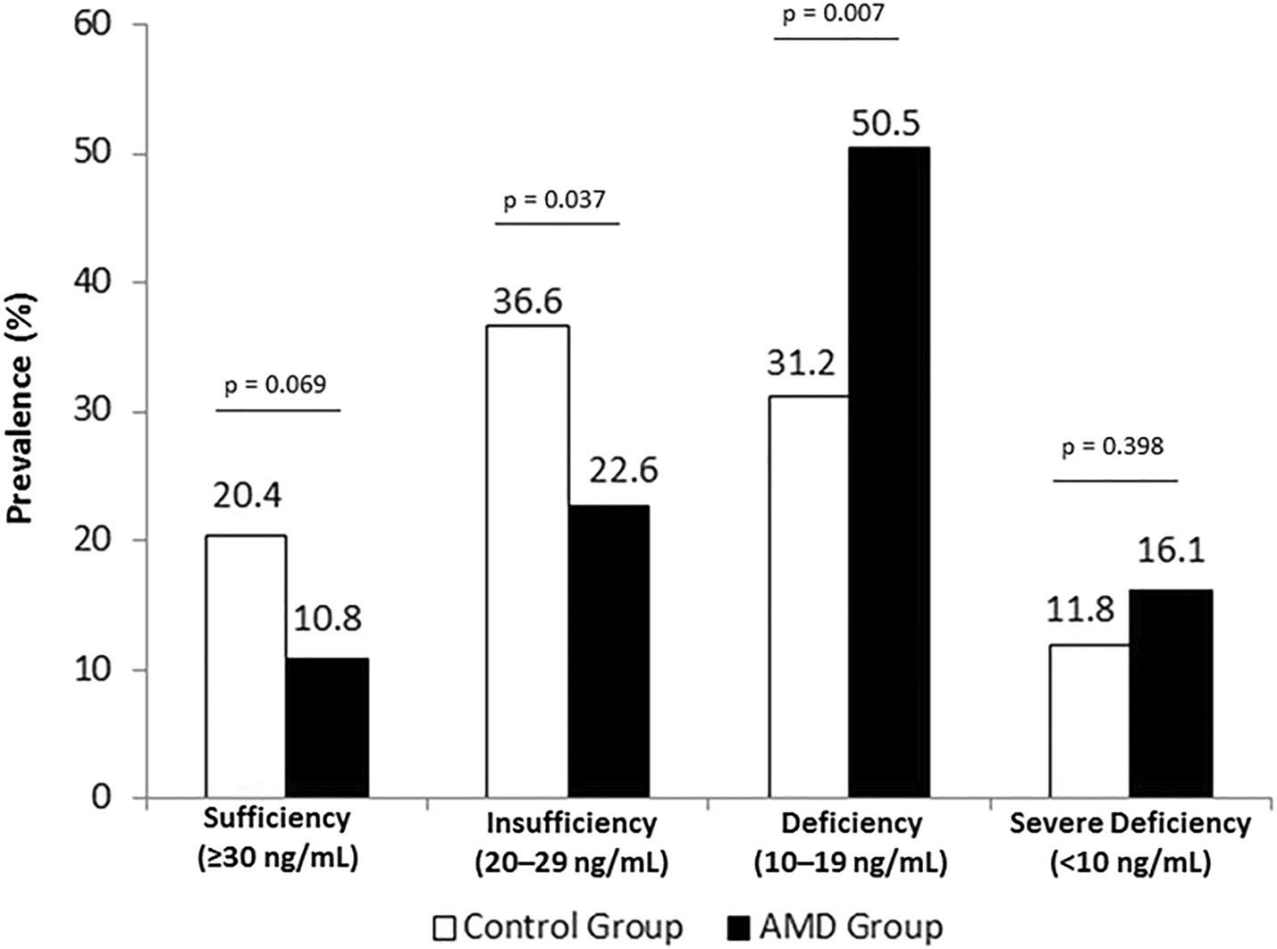
Prevalence of vitamin D sufficiency, insufficiency, deficiency and severe deficiency according to group

The prevalence of vitamin D deficiency was highest in the AMD group vs. control patients (47 (50.5%) vs. 29 (31.2%), respectively, p = 0.007). Rates of vitamin D severe deficiency were highest in the AMD group vs. control patients (15 (16.1%) and 11 (11.8%), respectively, p = 0.398). Instead, the prevalence of vitamin D insufficiency was highest in the control group vs. AMD group (34 (36.6%) vs. 21 (22.6%), respectively, p = 0.037). Rates of vitamin D sufficiency were highest in the control group vs. AMD group 19 (20.4%) vs. 10 (10.8%), respectively, p = 0.069). Fig. 3 and Table 1.

**Fig. 4:**
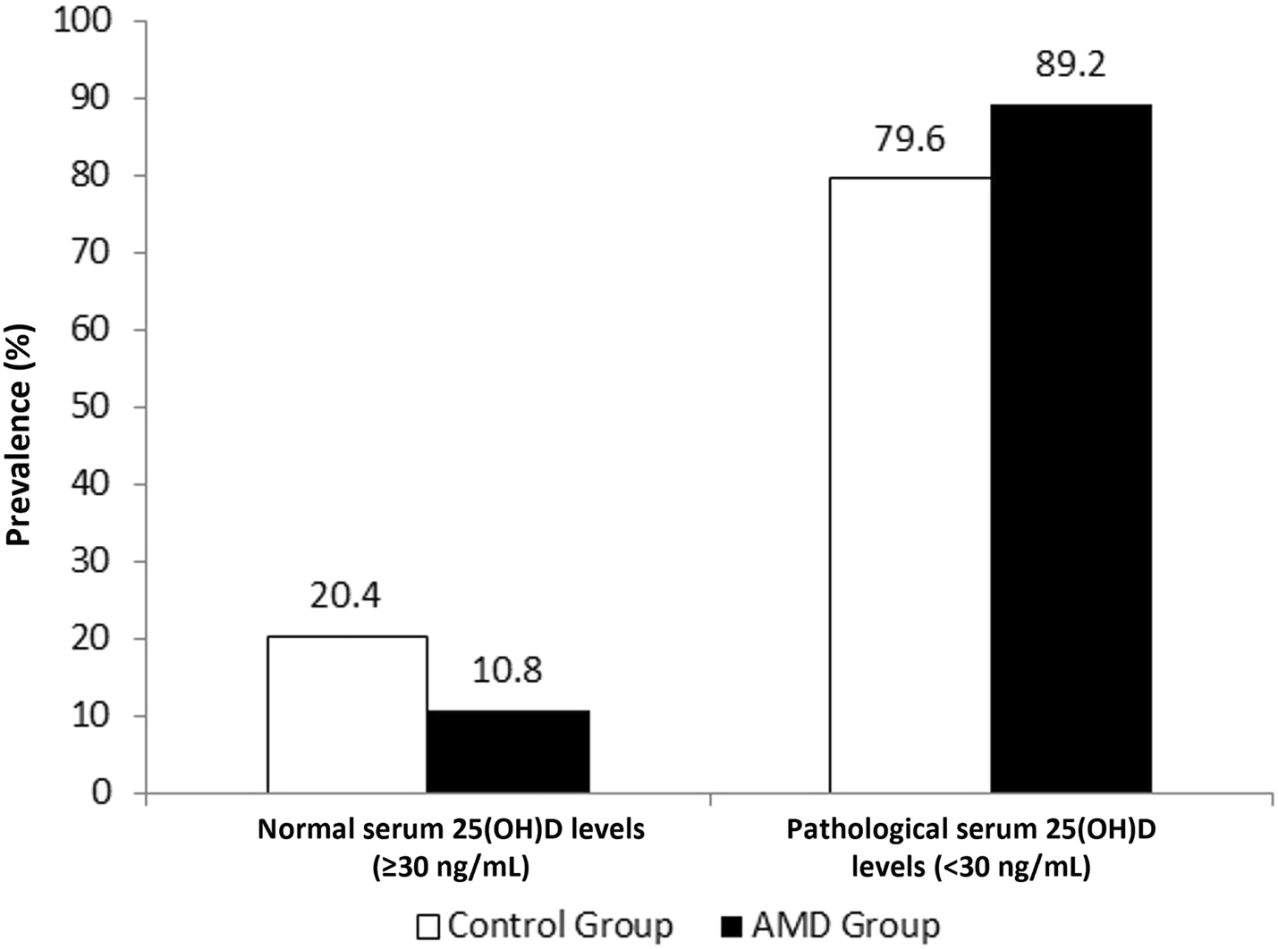
Prevalence of normal serum 25(OH)D levels (≥30 ng/mL) vs. abnormal serum 25(OH)D levels (<30 ng/mL) according to group

Eighty-three (89.2%) AMD patients had abnormal serum 25(OH)D levels (< 30 ng/mL) and 74 (79.6% in healthy patients. Ten (10.8%) AMD patients had normal serum 25(OH)D levels (≥ 30 ng/mL) vs 19 (20.4%) in healthy patients; p = 0.069. Fig. 4.

Finally in AMD group, abnormal serum 25(OH)D levels (< 30 ng/mL) was not associated with age (p = 0.858), gender (p > 0.999), BCVA (p = 0.274), IOP (p = 0.774), refraction (p = 0.542), state of the lens (p = 0.324), CFT (p = 0.673), AREDS2 supplementation (p > 0.999) or smoking status (p = 0.544) Table 2.

**Table 2:** Comparison between AMD patients with abnormal serum 25(OH)D levels and AMD patients with normal serum 25(OH)D levels

	Normal (n = 10)	Phatological (n = 83)	p
Age, mean ± SD	78.5 ± 5.1	79 ± 8.8	0.858
Gender			
Female	6 (60%)	51 (61.4%)	> 0.999
Male	4 (40%)	32 (38.6%)	
BCVA, median ± IQR	0.5 ± 1.05	1 ± 1.7	0.274
IOP, median ± IQR	14.5 ± 4	15 ± 4	0.774
State of lens			
Pseudophakic	3 (30%)	41 (49.4%)	0.324
Phakic	7 (70%)	42 (50.6%)	
CFT, Median ± IQR	251.5 ± 45	252 ± 63	0.673
AREDS suppl	4 (40%)	30 (36.1%)	> 0.899
Smoking status			
Current smoker	0	6 (7.2%)	0.544
Former smoker	2 (20%)	23 (27.7%)	
Non smoker	10 (80%)	54 (65.1%)	

*SD* standard deviation, *IQR* interquartile range

## Discussion

There is no consensus on the current status of the relationship between serum 25(OH)D levels and AMD. Two different meta-analyses show different results regarding the relationship between vitamin D and AMD [17, 18].

The first meta-analysis [17] supports the idea that age-related decrease in 25(OH)D concentration may expose to AMD onset and worsening and provides evidence that high 25(OH)D concentrations may be protective against AMD.

However, the second meta-analysis [18] shows there is no evidence to indicate an inverse association between serum vitamin D levels and any stages and subtypes of AMD risk.

One problem is defining what vitamin D value is normal and which is abnormal. Current guidelines suggest that 25(OH)D concentration values < 12 ng/mL are associated with an increased risk of osteomalacia, whereas 25(OH)D levels between 20 and 50 ng/mL appear to be safe and sufficient for skeletal health in the healthy general population [14]. It is not clear how or whether these guidelines should be considered with regard to individuals who have metabolic bone diseases, such as osteoporosis or primary hyperparathyroidism.

Different agencies and countries interpret 25(OH)D concentration levels in a different way. We chose recommendations for interpreting serum levels from the International Osteoporosis Foundation [15].

Itty et al. [19] defined vitamin D insufficiency, deficiency, and severe deficiency according to non-neovascular AMD (NNVAMD), neovascular AMD (NVAMD) and control group. The prevalence of vitamin D insufficiency, deficiency, and severe deficiency were all highest in the NVAMD vs. NNVAMD and control patients.

Day et al. [20] concluded that associations between vitamin D deficiency and a first diagnosis of NNVAMD and NVAMD were not statistically significant (p = 0.62 in NNVAMD group and p = 0.82 in NVAMD group). Moreover, our study did not show statistically significant differences between the different stages of AMD.

In a Korean population Kim et al. [21] showed that a high level of blood 25(OH)D was inversely associated with late AMD in men but not women. In another piece of Korean research Cho et al. [22] indicated that per 1 unit ng/mL increase in 25(OH)D the OR was 1.01 (95% CI 1–1.03, p = 0.179) in any AMD and 0.98 (95% CI 0.94–1.03, p = 0.501) in late AMD.

Golan et al. [23] in a study population comprised of 1,045 members diagnosed as having AMD, and 8,124 as non-AMD the mean ± SD level of 25(OH)D was 24.1 ± 9.41 ng/ml (range 0.8–120) for the AMD patients and 24.13 ± 9.50 ng/ml (range 0.0–120) for the controls, not statistically significant. They found no evidence for inverse association between 25(OH)D and AMD.

The serum 25(OH)D levels in our study are lower in the AMD and the control group, (17.12 ± 7.73 ng/mL and 21.51 ± 10.61 ng/mL, respectively). We found it difficult and inaccurate to compare the results of the different studies due to the different methods of measuring vitamin D, the differences between the study designs, and the different latitudes of the study populations.

Cutaneous vitamin D3 synthesis is diminished or absent at relatively high latitudes (> 35°N/S, particularly during the winter) by ecological factors that reduce ultraviolet B (UVB) penetration and by individual factors that limit cutaneous exposure to UVB, such as dark skin pigmentation, sun avoidant lifestyles, conservative clothing habits, and liberal use of sunscreen [24].

Our study is located in a region 39º north latitude in Spain, thus in a risk zone for vitamin D deficiency and the study was completed during the Covid-19 pandemic, so people could have had less cutaneous exposure to UVB and been able to synthesize less vitamin D although this would equally affect both of the studied groups.

Our study had some limitations. Firstly, the weaknesses of cross-sectional studies include the inability to make a causal inference. Secondly, the size of the samples in some groups was relatively small. Therefore, it was difficult to determine the exact correlation between vitamin D deficiency and AMD severity.

## Conclusion

In conclusion, this study shows that patients affected by AMD have lower vitamin D levels in comparison to healthy subjects. Further research is necessary to investigate the possible association between 25(OH)D levels and AMD.

## Data Availability

Access to SPSS data file may be provided at any point of time during the submission process.

## References

[1] Spaide RF, Jaffe GJ, Sarraf D (2020). Consensus nomenclature for reporting neovascular age-related macular degeneration data. Ophthalmology.

[2] Garcia-Layana A, Cabrera-López F, García-Arumí J (2017). Early and intermediate age-related macular degeneration: update and clinical review. Clin Interv Aging.

[3] Pennington KL, DeAngelis MM (2016). Epidemiology of age-related macular degeneration (AMD): associations with cardiovascular disease phenotypes and lipid factors. Eye Vis.

[4] Gheorghe A, Mahdi L, Musat O (2015). Age-related macular degeneration. Rom J Ophthalmol.

[5] Ferris FL, Wilkinson CP, Bird A (2013). Clinical classification of age-related macular degeneration. Ophthalmology.

[6] Kaarniranta K, Pawlowska E, Szczepanska J (2019). Can vitamin D protect against age-related macular degeneration or slow its progression?. Acta Biochim Pol.

[7] Holick MF (2018). Sunlight and vitamin D for bone health and prevention of autoimmune diseases, cancers, and cardiovascular disease1–4. Am J Clin Nutr.

[8] Gallagher JC (2013). Vitamin D and aging. Endocrinol Metab Clin North Am.

[9] Abokyi S, To C-H, Lam TT, Tse DY (2020). Central role of oxidative stress in age-related macular degeneration: evidence from a review of the molecular mechanisms and animal models. Oxid Med Cell Longev.

[10] Tohari AM, Zhou X, Shu X (2016). Protection against oxidative stress by vitamin D in cone cells. Cell Biochem Funct.

[11] Morrison MA, Silveira AC, Huynh N (2011). Systems biology-based analysis implicates a novel role for vitamin D metabolism in the pathogenesis of age-related macular degeneration. Syst Biol.

[12] Reins RY, McDermott AM (2015). Vitamin D: implications for ocular disease and therapeutic potential. Exp Eye Res.

[13] Layana A, Minnella A, Garhöfer G (2017). Vitamin D and age-related macular degeneration. Nutrients.

[14] Giustina A, Adler RA, Binkley N (2019). Controversies in vitamin D: summary statement from an international conference. J Clin Endocrinol Metab.

[15] Bouillon R (2017). Comparative analysis of nutritional guidelines for vitamin D. Nat Rev Endocrinol.

[16] Kan E, Kan EK, Yücel ÖE (2020). The possible link between vitamin D levels and exudative age-related macular degeneration. Oman Med J.

[17] Annweiler C, Drouet M, Duval GT (2016). Circulating vitamin D concentration and age-related macular degeneration: systematic review and meta-analysis. Maturitas.

[18] Wu W, Weng Y, Guo X (2016). The association between serum vitamin D levels and age- related macular degeneration: a systematic meta-analytic review. Invest Ophthalmol Vis Sci.

[19] Itty S, Day S, Lyles KW (2014). Vitamin D deficiency in neovascular versus nonneovascular age-related macular degeneration. Retina.

[20] Day S, Acquah K, Platt A (2012). Association of vitamin D deficiency and age-related macular degeneration in medicare beneficiaries. Arch Ophthalmol.

[21] Kim EC, Han K, Jee D (2014). Inverse relationship between high blood 25-hydroxyvitamin D and late stage of age-related macular degeneration in a representative Korean population. Investig Opthalmol Vis Sci.

[22] Cho B-J, Heo JW, Kim TW (2014). Prevalence and risk factors of age-related macular degeneration in Korea: the Korea national health and nutrition examination survey 2010–2011. Investig Opthalmol Vis Sci.

[23] Golan S, Shalev V, Treister G (2011). Reconsidering the connection between vitamin D levels and age-related macular degeneration. Eye.

[24] Roth DE, Abrams SA, Aloia J (2018). Global prevalence and disease burden of vitamin D deficiency: a roadmap for action in low- and middle-income countries. Ann N Y Acad Sci.

